# Effect of combined naltrexone and bupropion therapy on the brain’s functional connectivity

**DOI:** 10.1038/s41366-018-0040-2

**Published:** 2018-02-23

**Authors:** Gene-Jack Wang, Jizheng Zhao, Dardo Tomasi, Ehsan Shokri Kojori, Ruiliang Wang, Corinde E. Wiers, Elisabeth C. Caparelli, Nora D. Volkow

**Affiliations:** 1 Laboratory of Neuroimaging, National Institute on Alcoholism and Alcohol Abuse, Bethesda, MD USA; 2 College of Mechanical and Electronic Engineering, Northwest Agriculture and Forestry University, Yangling, Shaanxi China; 3 Department of Radiology, Henan Provincial People’s Hospital, Zhengzhou, Henan China; 4 Department of Psychology, Stony Brook University, Stony Brook, NY USA; 5 Neuroimaging Research Branch, National Institute on Drug Abuse, Baltimore, MD USA

## Abstract

**Background:**

The control of food intake in environments with easy access to highly rewarding foods is challenging to most modern societies. The combination of sustained release (SR) naltrexone and SR bupropion (NB32) has been used in weight-loss and obesity management. However, the effects of NB32 on the brain circuits implicated in the regulation of food intake are unknown. Here we used functional connectivity density (FCD) mapping to evaluate the effects of NB32 on resting brain FC.

**Methods:**

Thirty-six healthy women underwent magnetic resonance imaging (MRI) before and after 4-week treatment with NB32 (*n* = 16) or with placebo (*n* = 20). In each imaging visit, a 5-min resting-state functional MRI scan was conducted after 15 h of fasting. The FC of brain regions showing significant group effects on FCD were subsequently assessed using seed-voxel correlation analyses. We characterized the associations between FCD measures and craving control scores in the Control of Eating Questionnaire.

**Results:**

After NB32 treatment, the group showed lower local and global FCD than the placebo group in the right superior parietal cortex and lower local FCD in the left middle frontal gyrus. Seed-voxel correlation analysis for the right superior parietal cortex seed demonstrated higher positive FC with the dorsal anterior cingulate gyrus (ACC), bilateral insula, and left superior parietal gyrus and stronger negative FC with right inferior frontal gyrus and right superior parietal cortices for the NB32 than the placebo group. Further, the NB32 group showed a significant correlation between local FCD change after treatment in left middle frontal gyrus and craving control scores (*r* = 0.519, *p = *0.039).

**Conclusions:**

NB32 treatment decreased local and global FCD in superior parietal cortex and increased its connectivity with ACC (involved with saliency attribution), insula (interoception), and decreased local FCD in the medial prefrontal cortex (craving), which might underlie NB32 improved control over eating behaviors. ClinicalTrails.gov: NCT00711.

## Introduction

Although many signals that regulate food intake originate from internal sources that monitor the metabolic state of the body (i.e. leptin, insulin, ghrelin, PYY), variables other than nutritional needs also profoundly influence food intake. These include pleasurable sensory responses from food, emotional variables, and environmental factors [1]. Disruption in the sensitivity of the brain to these non-nutritional-related variables could result in excessive eating and obesity. Cognitive and affective processes are dysregulated in the eating behavior of obese subjects [2]. Particularly relevant are the rewarding and conditioned responses triggered by palatable foods. Functional magnetic resonance imaging (fMRI) studies have shown that obese subjects had strong blood oxygen level-dependent (BOLD) responses in brain regions related to motivation (dorsal striatum), salience attribution (orbitofrontal cortex), and taste information processing in insula while viewing pictures of high-caloric food (which act as powerful food cues that generate food craving [3, 4]). Obese subjects further showed greater disinhibition than non-obese subjects, which was associated with decreased activation in the anterior cingulate gyrus (ACC) when exposed to food cues [5].

Dopamine and endogenous opioids play critical roles in the control of eating behavior. Combination naltrexone-sustained release (SR) 32 mg and bupropion SR 360 mg (NB32) is a fixed-dose drug combination that was approved by Food and Drug Administration for treatment of obesity in 2014. The combination of naltrexone and bupropion has been demonstrated to result in greater weight loss compared to either agent alone [6]. The neurobiological mechanisms underlying the weight loss effects of NB32 are not fully understood but are thought to relate to bupropion stimulation of hypothalamic pro-opiomelanocortin neurons with downstream effects resulting in reduced food intake and increased energy expenditure. In addition, naltrexone results in a blockade consumption through the involvement of hypothalamic and brain stem mechanisms have been reported [7]. Naltrexone and bupropion in NB32 are also believed to reduce weight through synergistic activity in the mesolimbic reward system. Injection of either drug in the VTA of fasted mice leads to a decrease in food consumption, but together result in an even greater reduction than expected based on the individual components [8]. Our prior study using food-related cues and fMRI in women showed that NB32 therapy reduced hypothalamic reactivity to food cues while enhancing the activation of regions involved in self-control and internal awareness [9].

Previous studies have shown that chronic use of stimulants leads to neuroadaptation in the brain and interfere with resting functional connectivity (FC) between brain regions and networks implicated in eating behaviors [10, 11]. A resting-state fMRI study assessing FC in the brain of obese individuals showed alterations in networks involved in food reward and salience after an overnight fast [12]. However, the effects of NB32 treatment on FC or FC density (FCD) mapping are currently unknown. Low-frequency fluctuations (<0.1 Hz) in the BOLD signal are used to assess patterns of brain FC [13, 14]. The spontaneous BOLD fluctuations have been linked to low-frequency calcium oscillations that are markers of neuronal activity [15]. Whereas seed-voxel correlation approaches have been used to identify temporal synchrony between a specific brain region and the rest of the brain [16]. FCD has been used to assess local and global synchrony in the low-frequency BOLD fluctuations without the need to define specified seed regions. In the present study, we used FCD mapping to identify relevant regions for seed-voxel correlation analyses and to evaluate the effects of NB32 treatment on brain resting-state before and after 4 weeks of NB32 treatment. We hypothesized that NB32 would decrease FCD in brain regions involved in saliency, reward, and control processing and alter hypothalamic connectivity relative to a placebo (PLB) treatment.

## Methods and Procedures

### Subjects

The study protocol was approved by the Committee on Research Involving Human Subjects of Stony Brook University, New York. All participants provided written informed consent prior to study initiation. Subjects were screened carefully with a detailed medical history, physical and neurological examination, and urine toxicology for psychotropic drugs to ensure they were healthy at the time of the study and that they were not abusing drugs. Forty-six non-smoking subjects were included in the study with the following inclusion criteria: being female, right-handed, 18–45 years old and healthy; able to understand and give informed consent; and have a 27 ≤ body mass index (BMI) ≤ 40 kg/m^2^. Because most individuals who use pharmacotherapy for obesity are females, we only recruited female subjects to minimize variability. Exclusion criteria included obesity of known endocrine or genetic origin; history or presence of hepatic, renal, cardiovascular, or gastrointestinal diseases; type I or type II diabetes mellitus requiring pharmacotherapy; serious psychiatric illness; bulimia, or anorexia nervosa; history of alcohol or drug abuse or dependence (including nicotine); positive urine pregnancy test; head trauma with loss of consciousness >5 min; any medical condition that may alter cerebral function or contraindications for MRI.

### Study design

Subjects had two imaging visits, one at baseline and one after 4 weeks of treatment with NB32 or PLB. For women not using hormonal methods of contraception, the baseline visit was done in the follicular phase of the menstrual cycle. After the baseline visit, half of the participants were randomly assigned to the NB32 group and the other half to the PLB group. During the imaging visits, subjects underwent a 5-min resting-state fMRI scan under fasting conditions. Details on medication administration and preparation have been published previously [9]. Briefly, the naltrexone/bupropion combination consisted of daily doses of naltrexone SR 32 mg and bupropion SR 360 mg, combined in a trilayer tablet. Study drugs were escalated to full dose over 3 weeks. Both active and PLB tablets were blue, round, and identical in appearance. The subjects were instructed to maintain their usual eating and exercise habits throughout the study to minimize the impact of changes in nutritional status or body weight on brain activity. Study medication was withheld on the days of the scans until after completion of MRI scans. On the day prior to the imaging visits, the subjects were asked to have their last meal completed by 7 p.m. Subjects were informed that blood sugar levels would be checked during the study to help ensure that they refrained from eating. The scans were performed between 15 and 17 h after their last meal.

### Eating questionnaire acquisition

The Dutch Behavior and Eating Questionnaire (DBEQ) [17] and the Control of Eating Questionnaire (COEQ) [18] were obtained in each visit prior to the MRI scans. The DEBQ is a 32-item self-report questionnaire answered on 5-point Likert scales (1 = never, 5 = very often), and assesses three factors: emotional eating, externally induced and restrained eating. The DEBQ factors restrained eating [19] and emotional eating [20] have been associated with higher BMI, and the factor external eating has been shown to be related to greater vulnerability to food cues and impulsive eating that can result in higher BMI [21]. The COEQ consists of 21 items answered on 100 mm visual analog scales (0 = not at all, 10 = extremely) and assesses four components: craving control, positive mood, craving for sweet, and craving for savory. In this study, we used the craving control subscale [18], which composites of questions #1: How hungry have you felt? #9: During the last 7 days how often have you had food cravings? #10: How strong have any food cravings been? #11: How difficult has it been to resist any food cravings? #12: How often have you eaten in response to food cravings? and #19: Generally, how difficult has it been to control your eating? Lower craving control scores have been shown to be associated with stronger tendency to binge eat, and elevated disinhibition and susceptibility to hunger [18].

### MRI data acquisition

In each imaging visit, we conducted a 5-min resting-state fMRI scan (T2*-weighted single-shot gradient echo-planar imaging sequence in a 4-Tesla whole-body Varian/Siemens MRI scanner, TR/TE = 1600/20 ms, 4 mm slice thickness, 1 mm gap, 33 coronal slices, 64 × 64 matrix size, 3.1 × 3.1 mm resolution and 90° flip angle). Subjects kept their eyes open during the scan. Padding was used to minimize motion. Subject’s motion was monitored immediately after each fMRI run [22]. A “quiet” acquisition approach was used to minimize the interference effect of scanner noise during fMRI [23]. We also collected anatomical images using a T1-weighted three-dimensional modified driven equilibrium Fourier transform pulse sequence and a modified T2-weighted hyperecho sequence. These images were reviewed by a neurologist to rule out gross morphological abnormalities of the brain.

### FCD mapping

The functional images were realigned, normalized to the Montreal Neurological Institute space (voxel size: 3 × 3 × 3 mm^3^), 0.01–0.1 Hz band-pass filtered, and time points with excessive motion (framewise displacement > 0.5 mm) were removed. Nuisance signal fluctuations in white matter, cerebrospinal fluid, and the whole brain, as well as the six motion parameters (obtained from realignment step) and their temporal derivatives (total 18 regressors) were removed from data. Local and global FCD were then computed [24] for each image time series using a correlation threshold of *r* = 0.6 [25]. FCD brain mappings were smoothed with a Gaussian kernel of 6-mm full-width at half-maximum.

### Seed-voxel correlation

Regions-of-interest were defined as the clusters identified by SPM (group effect: PLB vs. NB32), and were selected as seed regions (8 mm radius sphere centered at the coordinates of the clusters peak) to calculate FC maps. The strength of the FC for each voxel was estimated using the Pearson's correlation coefficient between the average time- varying signal in the seed and each voxel in the brain voxels. The Fisher transform was used to convert correlation maps into normally distributed coefficient maps.

### Statistical analyses

A two-way ANOVA was implemented in SPSS22 model the effects of group (NB32, PLB) and time (baseline, 1 month later) on scores of DBEQ [17], sum craving control subscale, and question #19 within the COEQ [18]. A two-way ANOVA was implemented in SPM8 to model the effects of group (NB32, PLB) and time (baseline, 1 month later) on FCDM and seed-based FC. Statistical significance was based on family-wise error (FWE) corrections for multiple cluster-defining threshold of *P < *0.001. Pearson's correlation analyses were used to evaluate the link between FCD measures and BMI, DBEQ scores, and carving control scores.

## Results

Four subjects withdrew from the NB32 group due to adverse events such as vomiting, headache, and depression. Three subjects also withdrew from the PLB group: one with migraine, one lost to follow-up, and one who failed to comply with the protocol. In addition, MRI data from four subjects in the NB32 group had technical problems. Thus, data of 36 subjects (mean age ± SE: 31.19 ± 1.34 years old; BMI: 32.31 ± 0.65 kg/m^2^) who adhered to the treatment protocol and completed the fMRI studies (NB32: *n* = 16 and PLB: *n* = 20) were included in the study.

**Fig. 1:**
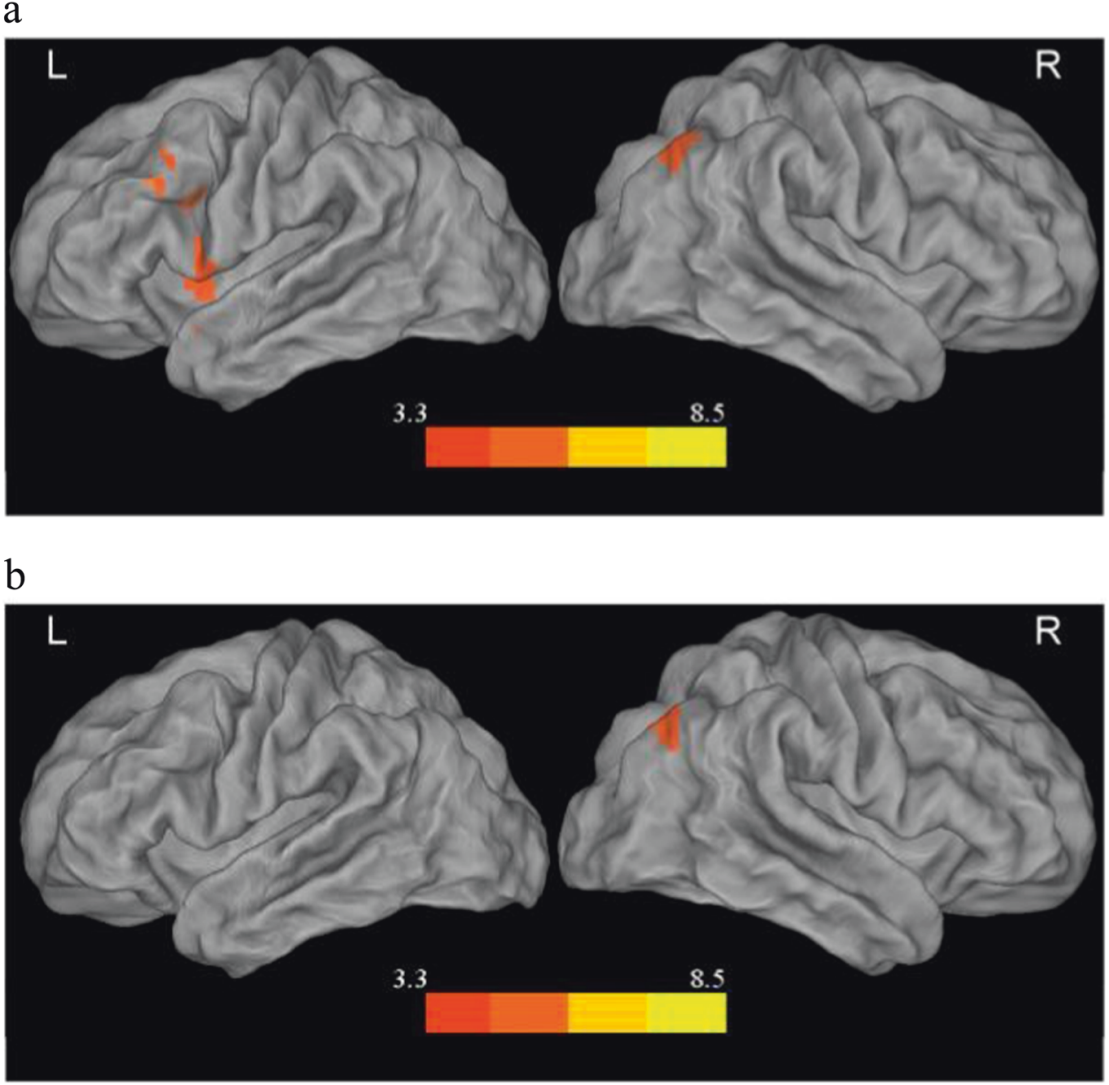
SPM results of brain regions with significant difference in functional connectivity density between NB32 group and PLB group (cluster level *P*_FWE_ < 0.05, *p* = 0.001, cluster size = 100) for local FCDM (**a**) and global FCDM (**b**)

At baseline, there were no significant differences between NB32 and PLB groups in demographic characteristics, eating behavior, and depression questionnaire scores, i.e., DBEQ, craving control subscale of COEQ (all *P* > 0.1; Table 1). There were no effects of 4 weeks of treatment on body weight, and DBEQ scores in either the NB32 group or the PLB group (all *P* > 0.05; Table 2). However, craving control scores significantly decreased after 4 weeks of treatment in both the NB32 and PLB group (F(1,34) = 9.693, *p* = 0.007). There were no significant group differences in global FCD (gFCD) or local FCD (lFCD) at baseline. After 4-week treatment, the NB32 group showed lower gFCD and lFCD in a region in the right superior parietal cortex in the boundary between BA39 and BA7 and lower lFCD in the left middle frontal gyrus (BA9/BA44/BA/13/BA6) than the PLB group (Fig. 1 and Table 3).

**Table 1:** Characteristics of the study subjects

	NB32 (*n* = 16)	PLB (*n* = 20)	
	Mean (SE)	Mean (SE)	*P* value(*t* test)
Age (years)	30.48 (1.97)	31.75 (1.88)	0.646
BMI (kg/m^2^)	32.72 (1.17)	31.98 (0.72)	0.582
DEBQ	76.50 (3.82)	72.80 (4.82)	0.566
Craving control (COEQ)	299.63 (27.06)	277.75 (25.42)	0.561

*NB32* naltrexone-sustained release (SR) 32 mg and bupropion SR 360 mg treatment group, *PLB* placebo group, *DEBQ* Dutch Eating Behavior Questionnaire, *COEQ* Control of Eating Questionnaire

**Table 2:** Behavioral scores before and after treatment

	Baseline, mean (SE)	1 month later, mean (SE)	Group × time	Main effect of time	Main effect of group
BMI					
NB32	32.7 (1.2)	32.5 (1.2)	F(1,34) = 0.24	F(1,34) = 2.47	F(1,34) = 0.26
PLB	32.0 (0.7)	31.9 (0.8)	*p* = 0.625	*p* = 0.125	*p* = 0.615
DEBQ					
NB32	76.5 (3.8)	74.1 (3.9)	F(1,34) = 3.97	F(1,34) = 0.08	F(1,34) = 0.02
PLB	72.8 (4.8)	76.1 (4.4)	*p* = 0.054	*p* = 0.778	*p* = 0.889
Craving control					
NB32	300 (27)	232 (32)	F(1,34) = 0.23	**F(1,34)** = **8.40**	F(1,34) = 0.15
PLB	278 (25)	229 (22)	*p* = 0.635	***p*** = **0.007**	*p* = 0.702
COEQ-Q19					
NB32	43.4 (5.9)	30.9 (5.1)	F(1,34) = 1.97	F(1,34) = 3.76	F(1,34) = 0.01
PLB	38.8 (6.3)	36.8 (5.4)	*p* = 0.170	*p* = 0.061	*p* = 0.939

*NB32* naltrexone-sustained release (SR) 32 mg and bupropion SR 360 mg treatment group, *PLB* placebo group, *DEBQ* Dutch Eating Behavior Questionnaire, *COEQ-Q19* generally, how difficult has it been to control your eating?. Bold values indicates statistically significant between NB32 and PLB

**Table 3:** Brain regions with significant difference between NB32 group and PLB group (cluster level *P*_FWE_ < 0.05, *p *= 0.001, cluster size = 100) for FCDM

Region	BA	Voxel	*Z*	MNI			Correlation analysis with craving control		Correlation analysis with Q19	
				*X*	*Y*	*Z*	NB32*	PLB	NB32*	PLB
							*r* (*p*)	*r* (*p*)	*r* (*p*)	*r* (*p*)
lFCDM, PLB vs. NB32										
Left middle frontal gyrus	9, 44, 13, 6	250	4.83	−45	23	31	**0.519 (0.039)**	0.333 (0.152)	**0.544 (0.026)**	0.308 (0.187)
Right superior parietal lobule	7, 40	465	4.73	30	−70	46	0.067 (0.806)	0.193 (0.416)	−0.309 (0.244)	0.408 (0.074)
gFCDM, PLB vs. NB32										
Right superior parietal lobule	7, 40	199	4.99	30	−79	46	0.089 (0.744)	−0.021 (0.928)	−0.388 (0.137)	−0.016 (0.946)

*NB32* naltrexone-sustained release (SR) 32 mg and bupropion SR 360 mg treatment group, *PLB* placebo group, *FWE* family-wise error, *MNI* Montreal Neurological Institute, *FCDM* functional connectivity density measure, *IFCDM* local FCDM, *gFCDM* global FCDM. Bold values indicate statistically significant

**Fig. 2:**
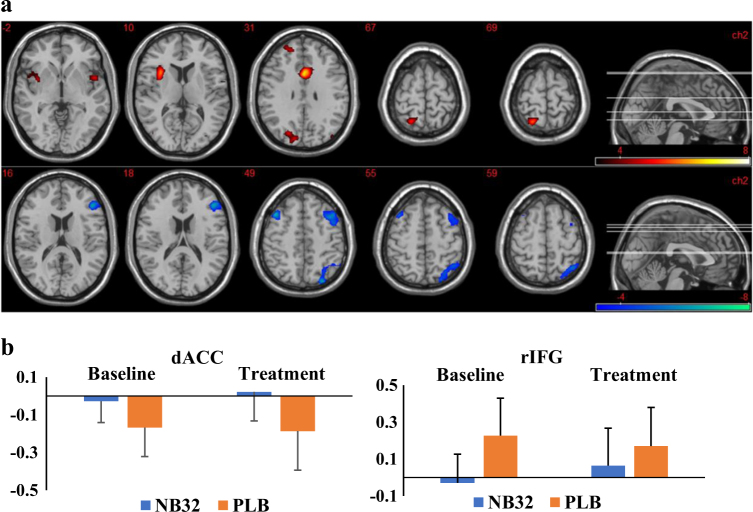
**a** SPM results of seed-voxel correlation analysis showing brain regions that have significant functional connectivity with superior parietal lobule; upper row: greater functional connectivity with superior parietal lobule in NB32 group than PLB group; lower row: stronger anticorrelation with right superior parietal cortices for the NB32 group than for the PLB group (cluster level *P*_FWE_ < 0.05, *p* = 0.001, cluster size = 100). **b** Functional connectivity (*z*-transform correlation) of superior parietal cortex at baseline after NB32 treatment with dorsal anterior cingulate cortex (dACC) and right inferior formal gyrus (rIFG)

Subsequently, we assessed FC of the brain regions showing significant group FCD effects using seed-voxel correlation analyses. The FC analysis for the right superior parietal cortical (BA the right superior parietal lobule seed demonstrated higher FC with the dorsal ACC, bilateral insula, and left superior parietal lobule for the NB32 than the PLB group (Fig. 2 and Table 4). The right superior parietal lobule also showed stronger anticorrelation with right inferior frontal and dorsolateral prefrontal cortices (DLPFCs) and with right superior parietal cortices for the NB32 group than for the PLB group (Fig. 2 and Table 4). The left middle frontal gyrus seed did not yield any significant functional correlation with any other brain regions after NB32 treatment.

**Table 4:** SPM results of seed-voxel correlation analysis

Region	BA	Voxel	*Z*	MNI		
				*X*	*Y*	*Z*
Positive correlation						
Dorsal anterior cingulate gyrus	24	184	5.65	0	11	31
Left anterior insula	48	230	5.15	−33	14	10
Left superior parietal lobule	7	242	4.77	−21	−61	67
Right anterior insula	48	123	4.26	51	5	−2
Negative correlation						
Right superior parietal lobule	7	348	−5.38	27	−82	49
Right inferior frontal gyrus	44	554	−5.16	45	32	16

Brain regions with significant functional connectivity difference between NB32 group and PLB group (NB32 vs. PLB, functional connectivity with the right superior parietal lobule cluster level *P*_FWE_ < 0.05, *p* = 0.001, cluster size = 100)

*NB32* naltrexone-sustained release (SR) 32 mg and bupropion SR 360 mg treatment group, *PLB* placebo group*MNI* Montreal Neurological Institute

**Fig. 3:**
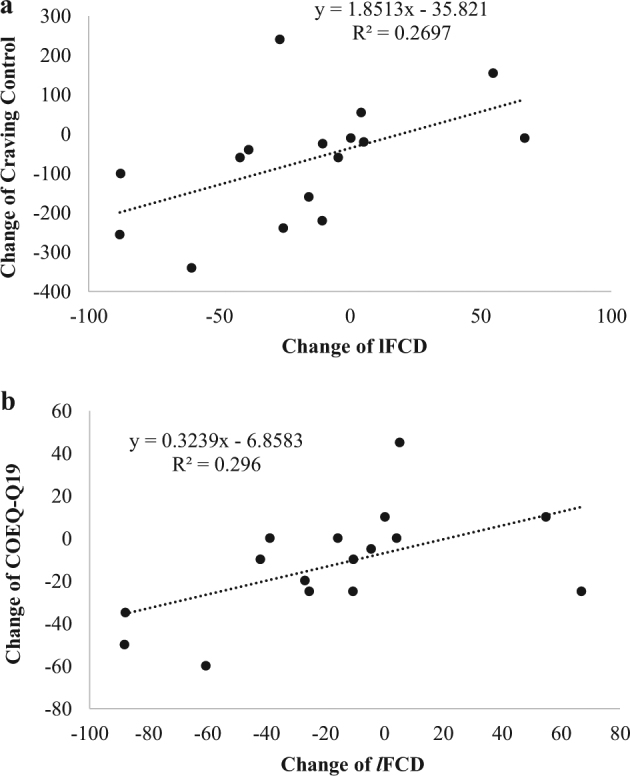
Correlation of changes between local functional connectivity density (lFCD) in left middle frontal gyrus and craving control scores in NB32 group (Fig. 3a, *n* = 16, *r* = **0.519**, *p* = **0.039**) and with scores of question #19 (generally, how difficult has it been to control your eating?) of COEQ in the NB32 group (Fig. 3b, *n* = 16, *r* = **0.544**, *p* = **0.026**)

However, there was a significant correlation between lFCD change in left middle frontal gyrus and the changes in craving control scores in the NB32 group (*r* = 0.519, *p* = 0.039), but not in the PLB group (*p* = 0.152) (Fig. 3 and Table 3). There was a significant correlation between lFCD change in left middle frontal gyrus and the changes scores of question #19 (how difficult has it been to control your eating?) within COEQ in the NB32 group (*r* = 0.544, *p* = 0.026), but not in the PLB group (*p* = 0.187) (Fig. 3b and Table 3). The correlation between lFCD changes in left middle frontal gyrus with changes in BMI (NB: *p* = 0.539; PLB: *p* = 0.958) or DBEQ (NB: *p* = 0.863; PLB: *p* = 0.711) were not significant (neither NS32 nor PLB). The correlation between lFCD changes in right superior parietal cortex with changes in BMI, COEQ, or DBEQ were not significant (neither NS32 nor PLB).

The FCD in the hypothalamus, which we a priori hypothesized would be sensitive to NB32 treatment, did not show significant group differences at baseline nor after 4 weeks of NB32 treatment.

## Discussion

After 4 weeks’ treatment significant differences emerged between the group treated with NB32 and the group treated with PLB. Specifically, NB32 was associated with a significant decrease in lFCD and gFCD in right superior parietal cortex (BA7/BA40) and in lFCD in the left middle frontal gyrus and with increased connectivity between the superior parietal cortex and the ACC, insula, and left superior parietal gyrus (BA7), while also showing increased anticorrelated connectivity with right inferior frontal gyrus.

Here we identified the superior parietal cortex as the region that showed consistent decreases in lFCD and gFCD with 4-week treatment with NB32. Though the superior parietal cortex is not typically associated with the neurocircuitry of obesity, a prior study had reported increased FC in the precuneus/posterior cingulate cortex (BA7/BA23) bilaterally in obese subjects [26]. Also, we had previously shown that NB32 treatment increased the activation of the right superior parietal cortex to the exposure of food-related cues as assessed with fMRI [9]. Here we expand this finding to also document using FC analyses that NB32 increases the connectivity of the right superior parietal cortex (BA7/BA4) seed with insula, which is a region involved with interoceptive awareness including perception of hunger [27] and with the left superior parietal cortex (BA7) while also increasing anticorrelated connectivity with right inferior frontal (including BA44 and BA46) [28], which are necessary for inhibitory control [29] and executive function [30]. This implicates the superior parietal cortex in integrating functions related to self-regulation and interoception. Additionally, the BA44 and right superior parietal cortices (BA7) are part of the ventral attentional control network [28, 31]. The ventral attention network has a crucial role in the ability to orient behavioral response when an endogenous or exogenous stimulus occurs unexpectedly. This network filters perceptual signals that allow identification of an appropriate behavioral response and simultaneously exert a protective activity against possible interferences with goal-oriented performance [32]. Bupropion is a norepinephrine-dopamine reuptake inhibitor that leads to increased extracellular norepinephrine and dopamine concentration and enhanced adrenergic and dopaminergic neurotransmission, which modulate attention networks [33]. Human studies using positron emission tomography have shown that bupropion treatment attenuates cue-induced increases in glucose metabolism in the anterior and posterior cingulate gyri [34]. Bupropion is reported to enhance smoking cessation by altering basal levels of dopamine through inhibition of dopamine reuptake while simultaneously modulating phasic dopamine release in the ventral striatum in response to smoking or smoking-related cues [35]. Combining the opioid antagonist naloxone with bupropion may have enhanced the modulation of the mesolimbic reward pathway [36]. The decrease of FCD in right superior parietal lobule and its FC with the attention network during resting-state after the NB32 treatment might help the obese subjects to modulate behavioral response to environmental stimuli, i.e., food cues.

After NB32 treatment, the right superior parietal lobule seed also demonstrated higher FC with the dorsal ACC, bilateral anterior insula, and left superior parietal cortex for the NB32 group than for the PLB group. The left anterior insula appeared relatively more connected with the right superior parietal lobule than the right as related to treatment efficacy. This functional asymmetry in the anterior insula has been reported in a recent study [37]. The ACC and the anterior insula are part of the salience network [38]. Saliency processing is altered in obese individuals [39]. MRI studies have shown ACC and bilateral anterior insula conjointly increase activation when cognitive tasks require effortful processing [40]. The ACC receives information from limbic and prefrontal regions to assess the salience of emotional and motivational information [41]. A brain fMRI study found that the activation of the dorsal ACC during food-related decision-making was positively correlated with self-reported cravings for high-fat foods [42]. Modulation of dopamine signaling in the ventral striatum via bupropion may alter reward signaling to the ACC and associated prefrontal regions, attenuating affective appraisal of cues and relative reward salience, thereby leading to a reduction in craving. Indeed, a prior fMRI study reported that bupropion decreased the activation of the ACC (as well as medial orbitofrontal cortex) when actively resisting craving compared to PLB and effect that was associated with a reduction in craving [43].

The NB32 group also showed lower FCD than the PLB group in the left middle frontal gyrus (lFCD) after 4 weeks of treatment. lFCD change in the left middle frontal gyrus correlated with craving control scores of COEQ in the NB32 group; even though craving control scores did not change after 1 month of treatment in the NB32 group. A clinical trial study with one participant showed significant body weight loss after an 8-week NB32 treatment and the body weight loss was correlated with craving control scores [44]. A subsequent study showed participants with the greatest improvement in craving control scores at week 8 exhibited a greater weight loss 1 year later [45]. The middle frontal gyrus in humans is part of the DLPFC, which is needed for executive function including, planning, goal implementation, drives, re-engages habits, craving, and behavioral control [2, 46, 47]. DLPFC is part of the ‘GO’ system that also includes ACC and orbitofrontal cortex [48]. Cue-induced activation of prefrontal cortical regions drives craving through functional connections with the striatum [49]. Both drug-addicted and obese individuals show abnormal activation of prefrontal cortical regions following cue-exposure, and this activation correlates with levels of elicited craving for drugs or food [49, 50]. In compulsive eating, this increased activation is thought to re-engage the basal ganglia circuitry involved in habitual overeating. Obese adolescent girls showed less activation of DLPFC when trying to inhibit high-calorie food images that was associated with reduced inhibitory control [51]. Adults who had greater DLPFC activation when instructed to resist the craving for food while viewing food images had better weight loss success following gastric bypass surgery [52]. Lower DLPFC response to high-calorie food images predicted greater ad libitum food intake over the next 3 days [53]. Consistent with this, a treatment approach directly targeting the DLPFC with transcranial direct current stimulation was shown to be effective in reducing craving for palatable food in binge eating women [54]. This effect of transcranial direct current stimulation may be effectively attenuating the cue-induced craving circuit modulated by the DLPFC [55]. The NB32 treatment induced lower FCD in left middle frontal gyrus, which might have contributed to the efficacy of control of eating [2], especially when the subjects were craving for food during resting state. Limitations: (1) We only recruited female subjects for this study since this is consistent with the population of most obesity pharmacotherapy studies. Therefore, these findings cannot be generalized to male obese individuals. (2) We used a relatively short resting state scan (5 min) in this study. However, a minimum of 10 min’ acquisition time has been suggested to obtain connectivity patterns across the entire brain with an optimal within-subject reproducibility [56]. Thus, replication of the current findings with longer resting state acquisitions are needed. (3) The participants enrolled in our study were within the same range of BMI as in a phase III randomized clinical trial study on NB32 treatment [44], and were given the same instructions to maintain eating and lifestyle habits [44]. The clinical trial study enrolled about 1500 participants and showed significant body weight loss after a 4-week NB32 treatment [44]. We, however, did not observe significant weight loss as an effect of treatment. It is likely that the sample size in the current study is insufficient to capture treatment effects on BMI.

## Conclusion

The marked decreases in FCD in the right superior parietal cortex and in left middle frontal gyrus after 4 weeks of treatment in the NB32 group suggests that the combined drug treatment approach affects the brain FC in obese and overweight women. NB32 therapy increased the FC of the parietal cortex with limbic regions involved in saliency and reward processing, and with reduced FCD in medial prefrontal regions involved in the control of craving. The FCD changes after the NB32 therapy might help to better understand neural mechanisms mediating the therapeutic benefits of NB32 in obesity.
